# Development of a Multi-Pulse Conductivity Model for Liver Tissue Treated With Pulsed Electric Fields

**DOI:** 10.3389/fbioe.2020.00396

**Published:** 2020-05-19

**Authors:** Yajun Zhao, Shuang Zheng, Natalie Beitel-White, Hongmei Liu, Chenguo Yao, Rafael V. Davalos

**Affiliations:** ^1^Department of Biomedical Engineering and Mechanics, Virginia Tech, Blacksburg, VA, United States; ^2^Bioelectromechanical Systems Laboratory, Virginia Tech, Blacksburg, VA, United States; ^3^State Key Laboratory of Power Transmission Equipment and System Security and New Technology, Chongqing University, Chongqing, China; ^4^School of Electrical Engineering, Chongqing University, Chongqing, China; ^5^Department of Electrical and Computer Engineering at Virginia Tech, Blacksburg, VA, United States

**Keywords:** pulsed electric field, electroporation, dynamic process, cumulative effect, tissue conductivity, tumor ablation, treatment planning

## Abstract

Pulsed electric field treatment modalities typically utilize multiple pulses to permeabilize biological tissue. This electroporation process induces conductivity changes in the tissue, which are indicative of the extent of electroporation. In this study, we characterized the electroporation-induced conductivity changes using all treatment pulses instead of solely the first pulse as in conventional conductivity models. Rabbit liver tissue was employed to study the tissue conductivity changes caused by multiple, 100 μs pulses delivered through flat plate electrodes. Voltage and current data were recorded during treatment and used to calculate the tissue conductivity during the entire pulsing process. Temperature data were also recorded to quantify the contribution of Joule heating to the conductivity according to the tissue temperature coefficient. By fitting all these data to a modified Heaviside function, where the two turning points (*E*_0_, *E*_1_) and the increase factor (*A*) are the main parameters, we calculated the conductivity as a function of the electric field (*E*), where the parameters of the Heaviside function (*A* and *E*_0_) were functions of pulse number (*N*). With the resulting multi-factor conductivity model, a numerical electroporation simulation can predict the electrical current for multiple pulses more accurately than existing conductivity models. Moreover, the saturating behavior caused by electroporation can be explained by the saturation trends of the increase factor *A* in this model. The conductivity change induced by electroporation has a significant increase at about the first 30 pulses, then tends to saturate at 0.465 S/m. The proposed conductivity model can simulate the electroporation process more accurately than the conventional conductivity model. The electric field distribution computed using this model is essential for treatment planning in biomedical applications utilizing multiple pulsed electric fields, and the method proposed here, relating the pulse number to the conductivity through the variables in the Heaviside function, may be adapted to investigate the effect of other parameters, like pulse frequency and pulse width, on electroporation.

## Introduction

Electroporation is a bioelectric phenomenon that employs high intensity, short duration pulsed electric fields (PEFs) to create reversible or irreversible defects in the cell membrane (Weaver and Chizmadzhev, 1996). Reversible electroporation (RE) occurs when the cell is able to recover from these defects and maintain high viability following treatment; alternatively, beyond a certain threshold, the process is irreversible and leads to cell death (termed irreversible electroporation, IRE) (Kotnik et al., 2012). Both RE and IRE are emerging oncological therapies (Mir et al., 1991; Yao et al., 2004; Davalos et al., 2005; Onik and Rubinsky, 2010). Electrochemotherapy (ECT) and gene electrotransfer (GET) utilize RE while IRE is mostly used as a non-thermal tumor ablation modality (Golberg and Yarmush, 2013; Haberl et al., 2013; Yarmush et al., 2013). Recently, another improved type of IRE termed high-frequency IRE (HFIRE) (Arena et al., 2011; Sano et al., 2015, 2018) was proved to ablate tumors with a benefit of mitigating muscle contraction and showed some promising results (Dong et al., 2018; DeWitt et al., 2019).

Therapeutic efficacy in either of these therapies is dependent on adequate coverage of the target area with electric fields capable of inducing electroporation; thus, clinicians are in need of tools capable of providing information regarding ablation outcome. In addition to post-treatment imaging using ultrasound, computed tomography (CT), or magnetic resonance imaging (MRI) (Onik and Rubinsky, 2010; Sommer et al., 2013; Ting et al., 2016), real-time evaluation of the ablation outcome has been proposed in recent studies (Ivorra et al., 2009; Zhao et al., 2018b). Pre-treatment visualization of the expected treatment zone also aids in achieving complete ablation (Edd and Davalos, 2007). However, the size and shape of the ablation are influenced by a number of factors, including electrode spacing and exposure, applied voltage, pulsing protocol, and tissue properties. These factors can be accounted for by using numerical models to predict the treatment zone (Neal et al., 2012). Pre-treatment planning models must account for the dynamic change in conductivity of the target tissue. This non-linearity is due to the nature of electroporation, in which low-resistance pathways form in cellular membranes within the target tissue. This change in resistance is dependent on the local electric field strength, causing a redistribution in the electric field (Davalos et al., 2004; Sel et al., 2005) and thus the expected treatment zone. Several studies have investigated this redistribution of the electric field caused by dynamic conductivity (Sel et al., 2005; Neal et al., 2012; Corovic et al., 2013; Zhao et al., 2018a), however, most of these studies only use the first treatment pulse to quantify the change of conductivity as a function of electric field. The effects induced by subsequent pulses are approximately reflected by the temperature rise in some studies (Garcia et al., 2010, 2011; Neal et al., 2012; Zhao et al., 2018a). In reality, electroporation causes cell membranes to become more permeable to ions, increasing the bulk conductivity. This effect can be enhanced as more pulses are applied. In this respect, electroporation can be viewed as a cumulative process, which means the pulse number should be incorporated into the conductivity model, making the numerical model more realistic. Langus et al. developed a model that described the dynamic conductivity as a function of electric field, temperature, and time (Langus et al., 2016). This model is complex, though it was well verified in reversible studies that employ eight pulses. In the case of multi-pulse IRE treatments, which employ dozens of pulses, to our knowledge, no study has developed a model that incorporates all three aspects.

In the present study, the often-used Heaviside function was employed to characterize the dynamic development of the conductivity as a function of electric field, temperature, and pulse number. Flat plate electrodes were used to deliver pulses to rabbit liver samples. The resulting voltage and current data were recorded to calculate the conductivity based on the constant shape factor of the flat plate electrodes, and the recorded temperature rise was used to quantify its contribution to the conductivity. The relationship between electric field strength and conductivity was then quantified using the Heaviside function, with the parameters as functions of pulse number. Finally, the dynamic conductivity model was used in a computational model employing the finite element method to predict the pulsed electrical current induced by multiple pulses. These results were compared to those obtained by using an existing conductivity model (Neal et al., 2012). The conductivity model presented in this study will be useful for developing a time-domain numerical model to study the electroporation process accounting for pulse number, electric field, and temperature.

## Materials and Methods

### Experimental Setup

**FIGURE 1:**
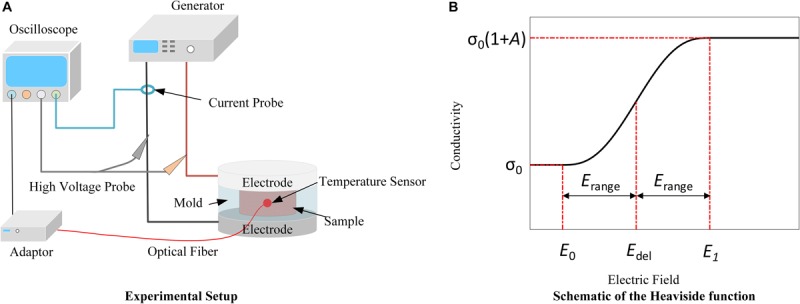
Schematics of the experimental setup **(A)** and the Heaviside function **(B)**. **(A)** Delivery of PEFs through flat plate electrodes enables conductivity measurements during multi-pulse treatments. Temperature is recorded by the fiber optic sensor to account for its contribution to the conductivity. **(B)** Due to electroporation, the tissue conductivity will change from its initial value (σ_0_) to the final value [σ_0_(1 + A)] with the transition zone from *E*_del_−*E*_range_ (*E*_0_) to *E*_del_ + *E*_range_ (*E*_1_).

Rabbit liver was obtained from the Experimental Animal Center of Chongqing Medical University. All procedures were in agreement with the Ethics Committee of Chongqing Medical University. The livers were acquired within 5 min of sacrifice and all tests were conducted within 1 h of organ procurement to prevent the effects of tissue degradation. Livers were placed in phosphate-buffered saline (PBS) in a cooler with ice before treatment to reduce the effect of metabolism on the tissue properties. Prior to the measurement, the liver samples were removed from the PBS and dried using paper tissue. Then they were cut into small pieces and filled in a 3D printed, polylactic acid (PLA) cylindrical mold as shown in Figure 1A. Every liver can be used to prepare about 6 samples, and 8 livers in total were used for this study. The mold features a slot for placement of a Luxtron m600 OEM fiber optic probe (FOT Lab Kit, LumaSense Technologies, Santa Clara, CA, United States), and the tissue temperature was sampled at 1 Hz. At the top center of the mold, there is a cylindrical space with a diameter of 1 cm and a height of 5 mm in which the tissue sample was placed. A total of 90 pulses were delivered for each experiment with an inter-pulse delay of 1 s and a pulse width of 100 μs. Applied electric field magnitude was varied for conductivity modeling: 100, 200, 400, 600, 800, 1,200, 1,400, 1,600, 1,800, 2,000, and 2,800 V/cm, while the magnitudes of 500, 1,000, 1,500, and 2,500 V/cm were used to verify the conductivity model. Every pulse magnitude was tested in 3 samples. All voltage and current data were recorded by an oscilloscope (WavePro 760Zi-A, Teledyne LeCroy Inc., New York, NY, United States) with a high voltage probe (PPE-5 kV) and a current sensor (Pearson 6600, Pearson Electronics Inc., Palo Alto, CA, United States). The temperature was recorded by the fiber optic sensor, and the adaptor was connected to the oscilloscope with TrueTemp software installed. The experimental setup is shown in Figure 1A. Since 100 μs pulses were applied in this study, the transient capacitive current was not considered, and the conductivity was calculated using the average voltage and current from the last 4 μs of each pulse (Supplementary Figure S1). The conductivity of the tissue was obtained by (1):

(1) $$\sigma =\frac{I\cdot L}{U\cdot S}$$

Where *U*(V) and *I*(A) are the averages of the last 4 μs of the recorded voltage and the current, respectively, *L*(m) is the thickness of the sample and *S*(m^2^) is the cross-sectional area of the sample.

### Dynamic Model of Conductivity

At the end of each consecutive pulse, the calculated conductivity at different electric fields can be described. For each of the 90 pulses, a Heaviside function (2) describing conductivity as a function of electric field was fit to the experimental data (Zhao et al., 2018a).

(2) $$\sigma \,\left(\left|E\right|,T\right)=\sigma_{0}\cdot \left(1+A\cdot \mathrm{flc2hs}\,\left({\text{E–E}}_{\text{del}},E_{\text{range}}\right)+\alpha \cdot \left({\text{T–T}}_{0}\right)\right)$$

Here, σ_0_(S/m) is the initial conductivity of the tissue before treatment, *A* is the increase factor of the conductivity after treatment, *flc*2*hs* is the Heaviside function, *E*_del_(V/m) and *E*_range_(V/m) define the transition zone (Figure 1B); and the exact expression of the function can be found in the Supplementary Material S2. In (2), the value of σ_0_ ⋅ *A*⋅*flc2hs* (*E*−*E*_del_, *E*_range_) changes from 0 to σ_0_⋅*A* with the transition from *E*_del_−*E*_range_, defined as *E*_0_(V/m), to *E*_del_ + *E*_range_, defined as *E*_1_(V/m). This term is used to describe the contribution of electroporation to conductivity. More information about these parameters can be found in Zhao et al. (2018a). *E* is the electric field magnitude (V/m), and α(1/°C) is the coefficient that reflects the conductivity change due to temperature variation. Here, the value of 2% was chosen from the literature (Duck, 2013). T(°C) is the real-time tissue temperature, and T_0_ is the initial temperature before treatment, set to 24°C. The temperature data for fitting were from our measurements. Therefore, the three terms on the right side of the equation quantify the tissue’s intrinsic electrical properties (σ_0_), electroporation effect (σ_0_ ⋅ *A*), and temperature effect [σ_0_ ⋅α(T−T_0_)], respectively.

After fitting the conductivity and electric field data to the Heaviside function, the value of the main variables (*A__*i*_*, *E*_del__*_*i*_*, and *E*_range__*_*i*_*, where *i* is the pulse number from 1 to 90) in the conductivity model for each pulse number was determined. *E*_0_ and *E*_1_ are the two turning points of the curve, indicating the points at which the conductivity begins to increase and begins to saturate, respectively. The change of these two variables with pulse number is intuitive; with more pulses applied, the tissue would be easier to electroporate, which means lower electric fields (turning points) can induce the conductivity change resulting from electroporation. Therefore, our hypothesis is that the asymptote of *E*_0_, the electric field where tissue conductivity starts to change, approaches zero when the pulse number tends to infinity. According to analysis from the Supplementary Material S3, *E*_1_ does not change much with pulse number (< 5%). Thus, *E*_0_ was chosen as the fitting parameter in lieu of *E*_del_ and *E*_range_. The following data fitting was conducted to establish models for *E*_0_ and *A*, which are treated as functions of pulse number. This process served as a bridge to build the relationship between the pulse number and the tissue conductivity variation caused by PEFs. According to the trends of these variables, the symmetrical sigmoidal function (3) was used to describe the relationship between *A* and the pulse number (Lin et al., 2012), while the change of *E*_0_ with pulse number was reflected by Equation (4).

(3) $$A=a_{1}\left(1-{\left(1+N\cdot b_{1}^{-1}\right)}^{-c_{1}}\right),\left(a_{1},b_{1},c_{1}>0\right)$$

(4) $$E_{0}=a_{2}N^{-1}+b_{2}e^{-c_{2}\,N},\left(a_{2},b_{2},c_{2}\geq 0\right)$$

Here, *N* is the pulse number, and *a_(__1,__2__)_*, *b_(__1,__2__)_*, and *c_(__1,__2__)_* are coefficients determined by data fitting. MATLAB (R2018b, MathWorks) and Excel (Microsoft Office Professional Plus 2019) were used to perform the curve fitting and data analysis, respectively.

### Validation of the Conductivity Model

The pulse parameters with varied electric field magnitude (500, 1,000, 1,500, 2,500 V/cm) were used to verify the conductivity model by comparing the experimental current outputs and the numerical results. Two cylinders with diameters of 2 cm and thicknesses of 2 mm were built in COMSOL Multiphysics (v.5.4, Stockholm, Sweden) as two flat plate copper electrodes. Another cylinder with 2 cm diameter, 5 mm height and a 1 cm diameter through-hole in the center was constructed to represent the 3D printed mold used in experiments. The tissue sample was modeled as a cylinder with a diameter of 1 cm and a height of 5 mm. The Laplace equation was used to solve for the electrical potential, and the electric field distribution was obtained by taking the gradient of the electrical potential:

(5) ∇⋅(σ⁢(E,N,T)⁢∇⁡φ)=0

(6) E=-∇⁡φ

Here, σ (*E, N, T*) is the tissue conductivity which is a function of electric field, pulse number, and temperature; *E* is the electric field, *N* is the pulse number and *T* is the temperature.

Temperature in the finite element model was calculated by a modified heat conduction equation including the Joule heating term:

(7) $$\nabla \cdot \left(k\,\nabla T\right)+\frac{\sigma \,{\left|\nabla \varphi \right|}^{2}\cdot d}{\tau }=\rho \,c_{p}\,\frac{\partial T}{\partial t}$$

Here, *k* is the thermal conductivity of the tissue, *c*_p_ is the tissue heat capacity, ρ is the tissue density, and σ|∇⁡φ|^2^ is the Joule heating term which was scaled according to the pulse width *d* divided by the period τ, averaging the heating over the entire treatment (Neal et al., 2012). Since the tissue sample and the contacting area between the electrodes and the tissue are relatively small, the generated Joule heating will not have a significant effect on the outside boundary of the electrodes and the mold. Therefore, the outside boundary of the electrodes and the mold was set to room temperature, 24°C. The blood perfusion and metabolic heat were not included since the studies were conducted *ex vivo*. Parameters values used here are shown in Table 1.

**TABLE 1:** Material properties used for the simulation.

**Material**	**Parameter**	**Value**	**Unit**	**References**
Liver	ρ, density	1,079	kg/m^3^	Zhao et al., 2018a
	*c*_p_, heat capacity	3,540	J/kg/K	Zhao et al., 2018a
	k, thermal conductivity	0.52	W/m/K	Zhao et al., 2018a
PLA	ρ, density	1,252	kg/m^3^	Farah et al., 2016
	*c*_p_, heat capacity	1,590	J/kg/K	Farah et al., 2016
	k, thermal conductivity	0.11	W/m/K	Farah et al., 2016
	σ, electrical conductivity	1e–16	S/m	Zenkiewicz et al., 2011
Electrode (Copper)	ρ, density	8,960	kg/m^3^	Comsol Material Library
	*c*_p_, heat capacity	385	J/kg/K	Comsol Material Library
	k, thermal conductivity	400	W/m/K	Comsol Material Library
	σ, electrical conductivity	5.998e7	S/m	Comsol Material Library

To determine the impact of incorporating multiple pulse tissue conductivity parameters in the proposed model, a comparison was made to the existing conventional conductivity model, σ(*E, T*), where the tissue conductivity is determined following the first pulse. From the proposed model, the conventional conductivity model can be obtained by setting the pulse number, *N*, to 1 pulse; subsequent increases in tissue conductivity are then attributed solely to Joule heating effects (Garcia et al., 2010, 2011; Neal et al., 2012; Zhao et al., 2018a). The normal current density over the middle plane of the tissue which was in parallel with the electrodes was integrated to obtain the total current delivered to the tissue (Equation 8), which was used to compare the agreement of the results between simulation and experiments.

(8) $$I_{s}=\int \int \text{ec}.\text{normJ}$$

Here, ec.normJ is the magnitude of current density perpendicular to the middle plane of the tissue which is in parallel with the flat plate electrodes. *I*_s_ is the calculated current that is used to compare with the experimental results.

The error between the experimental data and the simulated results was described by the root-mean-square error (RMSE) (Barnston, 1992):

(9) $$\mathrm{RMSE}={\left[\sum_{i=1}^{n}{\left(f_{i}-y_{i}\right)}^{2}/n\right]}^{1\,/\,2}$$

Here, *n* is the total applied pulse number. *f*_*i*_ is the simulated results, and *y*_*i*_ is the data from the experiments. A smaller RMSE indicates lower error between the simulation results and the experimental results, demonstrating a more accurate prediction.

## Results and Discussion

**FIGURE 2:**
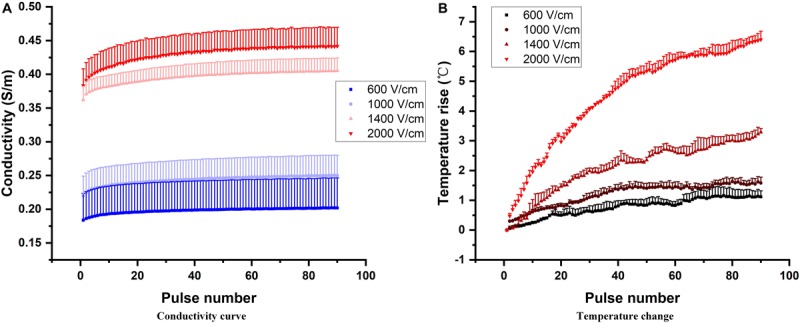
Conductivity dependency on pulse number at different electric field magnitudes **(A)**. Increasing electric field magnitudes cause a vertical shift in multi-pulse conductivity. Pulse number also has an effect on the dynamic behavior of the conductivity, as reflected in the non-linear increase during the first few dozen pulses and nearly linear increase with the subsequent pulses. The temperature rise at those corresponding pulse parameters is shown **(B)**.

In this study, we applied 90, 100 μs pulses to capture the dynamic change of tissue conductivity caused by electroporation. The initial transient tissue response, mostly capacitive current, to the pulses was not included in calculating the tissue conductivity change caused by electroporation (Neal et al., 2012). The tissue conductivity during each pulse was quantified by the end of each voltage and current pulse (details can be found in the Supplementary Material S1). The conductivity and temperature change with pulse number at different electric fields is shown in Figure 2 (only 4 magnitudes are shown). With higher electric field and more pulses applied, the tissue conductivies and temperature rise increase. The dynamic behavior of each conductivity curve in Figure 2 can be divided into two parts: first, the non-linear change happens during the first few dozen pulses, which mainly reflects the cumulative effect of multiple pulses on electroporation. However, this non-linear behavior could not be captured by the conventional conductivity model which was based solely on the first pulse (the electroporation effect), and the temperature rise (cumulative effect of multiple pulses). After the initial non-linearity, the tissue conductivity seems to increase linearly with pulse number, potentially indicating that the electroporation effect saturated, and the increase in conductivity is likely attributed to increases in temperature. This non-linear process might provide insight into the electroporation process in tissue. In this study, we quantified this non-linear process by creating mathematical models.

### Data Fitting Results

**FIGURE 3:**
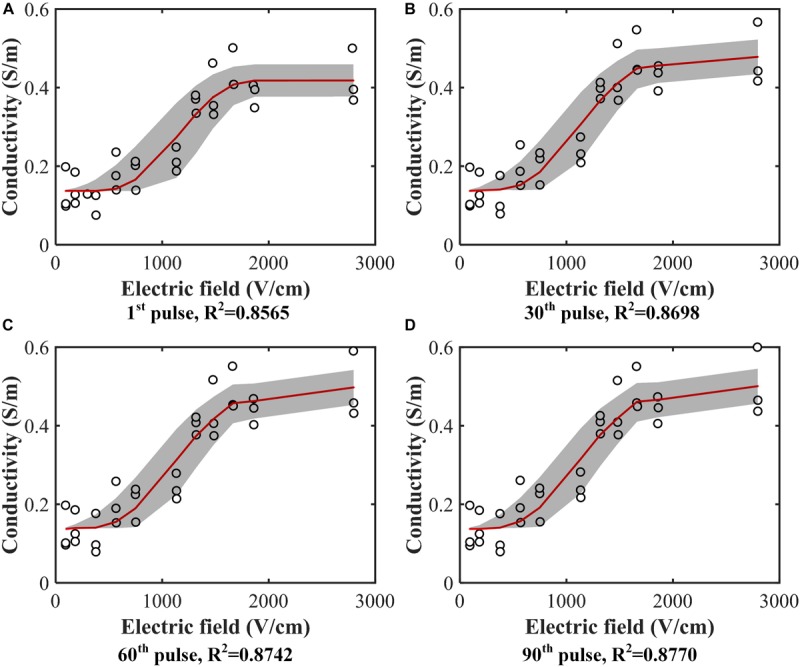
Conductivity data (hollow circle) from experiments with the fitted curves [red solid line, Equation (2)]. Conductivity values were calculated at each electric field magnitude at the **(A)** 1^st^ pulse, **(B)** 30^th^ pulse, **(C)** 60^th^ pulse, and **(D)** 90^th^ pulse. Curves were fitted for each case. The gray band is the 95% confidence interval of the fitting. All the fitting results can be found in the Supplementary Material S4.

The tissue conductivity change during electroporation is a suitable indirect method to help us further understand the dynamic process of electroporation (Sel et al., 2005; Neal et al., 2012; Zhao et al., 2018a). In this study, all treatment voltage and current pulses were recorded to obtain the tissue conductivity change during the entire treatment process. Figure 3 shows the tissue conductivity at different electric field strengths at 4 specific pulse numbers. The experimental data were fitted using (2) at each pulse number, and the fitted curves are shown as the solid line in Figure 3. The coefficients of determination, R^2^, for all 90 curves (data were shown in the Supplementary Material S4) were above 0.85 which indicates acceptable data fitting accuracy. As more pulses are applied, the conductivities at higher electric fields were a little upward shift rather than flat which was caused by the temperature rise. At lower electric field strengths, the tissue was not electroporated, and the conductivity at these fields was the initial conductivity of the tissue. After data fitting, the results showed that the initial conductivity for different pulse numbers did not change significantly, as expected. The initial conductivity for (2) was determined as the average of all the initial values of conductivity for different pulse numbers, calculated to be 0.137 S/m in this study.

**FIGURE 4:**
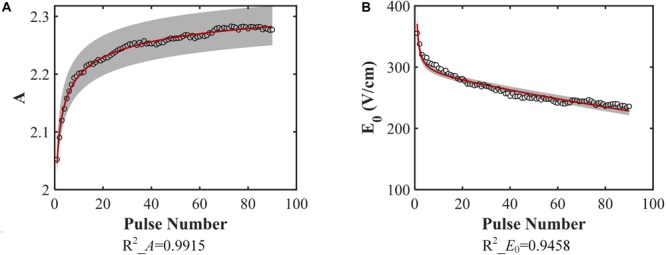
Model parameters. **(A)** *A*, **(B)** *E*_0_ change with increasing pulse number and the data fitting results [**A:** Equation (3), **B:** Equation (4)]. The coefficients of determination of the fitting are given below each figure. The gray band is the 95% confidence interval of the fitting. They don’t include 0, indicating that they are significantly different from 0.

After fitting the conductivity data, the change in the variables (*A*, *E*_0_) with increasing pulse number was obtained, as shown in Figure 4. Each hollow circle in the figure represents a value of *A* or *E*_0_ determinted by the data fitting using Equation (2) for a specific pulse number. Therefore, there are 90 values of *A* and *E*_0_, respectively. The coefficients of determination *R*^2^ for each curve fit are shown below each figure, which indicates successful fitting results for these two parameters.

The obtained equations for these two parameters are shown below:

(10) $$A=2.3919\times \left(1-{\left(1+N\cdot 0.0018^{-1}\right)}^{-0.2802}\right)$$

(11) $$E_{0}=8.0861\times 10^{3}\,N^{-1}+2.9056\times 10^{4}\,e^{-0.0027\,N}\,\left(V/m\right)$$

where *N* is the pulse number. From these equations, the top and bottom limits for each parameter are readily identifiable. Since *N* must be a non-negative integer, the lower limit for these parameters will be obtained at *N* = 0, which means no pulses are applied. When *N* = 0, *A* = 0, and *E*_0_ tends to infinity. The upper limit can be obtained when *N* tends to infinity; in this case, *A* = 2.3919, and *E*_0_ tends toward 0.

### Validation of Outcome and Comparison With Conventional Conductivity Model

**FIGURE 5:**
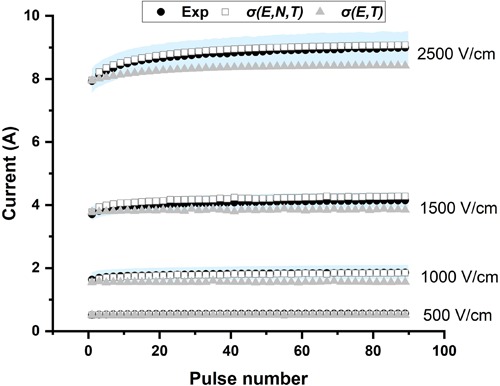
The presented conductivity model [σ(*E*, *N*, *T*)] predicts the experimental current more accurately than the conventional model [σ(*E*, *T*)]. The experimental data are plotted as the mean (black solid circle) and standard deviation (light blue shadow).

The conductivity model in this study, which is a function of electric field, pulse number, and temperature, can be obtained by inserting (10–11) into (2). To verify the model, the experimental results of the selected pulse parameters were compared to the numerical results. To compare the conductivity model proposed in this study, σ(*E*, *N*, *T*), and the conventional conductivity model, σ(*E*, *T*), the electrical current calculated using these two models in COMSOL and the experimental data at different pulse numbers are shown in Figure 5. As expected, the current obtained from the conventional model was almost linear with increasing pulse number. The non-linear portion during the first 20–30 pulses cannot be replicated by the conventional conductivity model but is captured using the σ(*E*, *N*, *T*) model. These results show that the conductivity model developed herein can describe the electroporation process for multiple pulses more accurately than σ (*E*, *T*).

**FIGURE 6:**
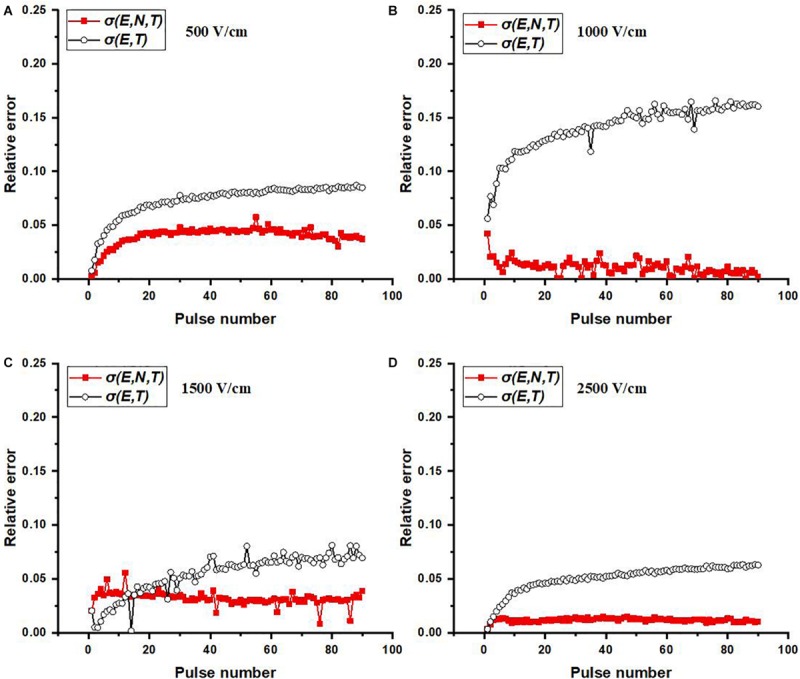
Relative errors at each pulse number for varied electric field magnitudes: **(A)** 500, **(B)** 1,000, **(C)** 1,500, and **(D)** 2,500 V/cm. Relative error at each pulse number is calculated by | simulated value – averaged experimental value| /averaged experimental value.

Figure 6 presents the relative errors which were calculated at each point (pulse number) using the simulated data and the averaged experimental data (black solid circle in Figure 5). Table 2 shows the RMSE values for four electric field magnitudes which are used to verify the conductivity model. From Figure 6, the relative errors for σ(*E*, *N*, *T*) are all below 0.05. In Table 2, RMSEs for σ(*E*, *N*, *T*) are all smaller than those of σ(*E*, *T*). Both the results show that the predicted data using σ(*E*, *N*, *T*) has lower errors than the data obtained by σ(*E*, *T*). At lower electric field magnitude, both of these models have low RMSE values; this is likely due to a lesser extent of tissue electroporation. Therefore, cumulative electroporation effects are not obvious. For higher electric field magnitude, the error of σ(*E, T*) increases sharply after the first few dozen pulses since the cumulative effect is not included.

**TABLE 2:** Root-mean-square error between simulated and experimental results based on two conductivity models.

**Conductivity model**	**RMSE**			
	**500 V/cm**	**1,000 V/cm**	**1,500 V/cm**	**2,500 V/cm**
σ(*E*, *N*, *T*)	0.0592	0.3724	0.4593	0.8404
σ(*E*, *T*)	0.0839	0.5800	0.5678	1.1423

### Parameter Variation of Conductivity Model

In this study, the Heaviside function with a continuous second derivative was used to describe the change of tissue conductivity with different pulse parameters. Excluding the effect of the temperature, the conductivity variation caused by electroporation will change from σ_0_ to σ_0_(1 + *A*). From Figure 4A, with the application of multiple pulses, *A* will change from its initial value to its asymptote with a value of 2.3919, which indicates that the conductivity change resulting from electroporation will be saturated at the value of 0.465 S/m for rabbit liver tissue. This conclusion is also consistent with previous work indicating that when tissue is completely electroporated, the conductivity will cease changing due to electroporation (Ivorra and Rubinsky, 2007; Maor et al., 2009). Some studies reported that the ablation zone of IRE tended to be saturated after dozens of pulses (Miklovic et al., 2017) which could reflect the saturation behavior too.

**FIGURE 7:**
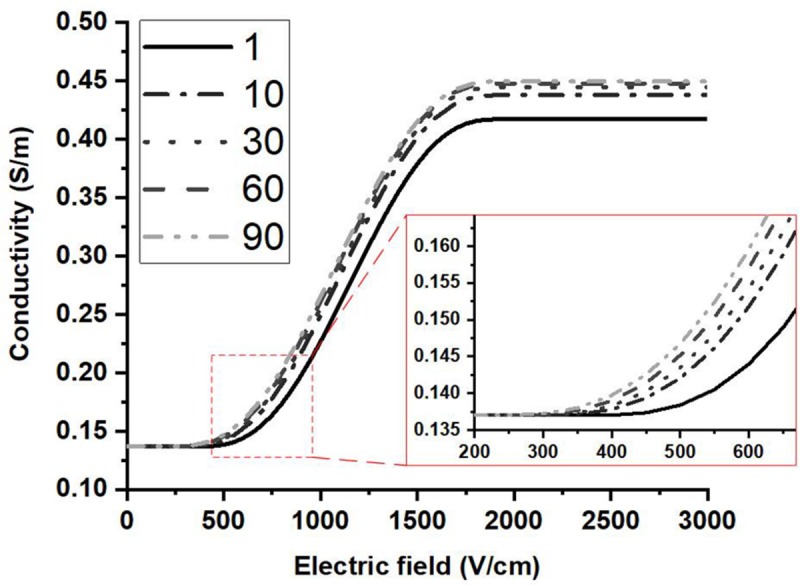
Conductivity vs. electric field curves are shown at different pulse numbers based on the proposed conductivity model. The contribution of the temperature change to the conductivity was not included. The plateau of each curve saturates after a few dozen pulses.

The conductivity curve excluding the temperature effect can clearly reflect the electroporation process, as shown in Figure 7. After the first few dozen pulses, the plateau of each curve, σ_0_(1 + *A*), begins to saturate, indicative of a change in *A* with pulse number. The first inflection point moves to a lower value with a higher pulse number which can also be seen from the change in *E*_0_. The variation of the inflection point indicates that lower electric fields can induce tissue conductivity changes when more pulses are applied, which means that the tissue will be easier to electroporate with more pulses applied. This might be an explanation of the observation that the electric field threshold for IRE was lower with more pulses applied (Bonakdar et al., 2015; Bhonsle et al., 2016; Zhao et al., 2018a).

Figure 5 shows that the model proposed in this study can describe the electroporation process more accurately than the typically used conductivity model. The proposed conductivity model which includes the effect from electric field, pulse number, and temperature can help us understand the electroporation process at the tissue level, and would result in more precise treatment planning. In this study, a non-linear process is obvious during the first few dozen pulses (∼30 pulses); however, this does not necessarily ensure that ∼30 pulses can induce irreversible electroporation. Since the recovery process was not evaluated in this study, more pulses are most likely needed to maintain the electroporation effect and create a relatively stable irreversible electroporation outcome. Future work should pay more attention to the recovery process of the tissue after electroporation. Incorporating these effects will allow for the optimization of pulse parameters (pulse magnitude, pulse number, et al.) which need to be applied to induce expected irreversible electroporation effects.

The success of IRE treatment depends on a good treatment planning of IRE. Olivier Gallinato et al. (2019) proposed a numerical workflow of IRE based on the real clinical situation (placement of electrodes and the response current) which should be the future trend to make patient-specific treatment planning. The significant part of making treatment planning is the calculation of the electric field distribution which is affected by the tissue conductivity distribution during the treatment. Damien Voyer et al. (2018) recently built a dynamic model of tissue electroporation based on the equivalent circuit approach at the tissue level. By their model, the electroporation models at cell and tissue levels can be linked, which is good for us to understand the electroporation process in tissue. This model was validated by the first pulse; applications with more pulses, such as 90 pulses for IRE, still need to be further studied. In the present study, the conductivity model can be used to predict the current for 90 pulses accurately. However, this is only valid for the current at the end of the pulse rather than the entire pulse.

In the chosen conductivity model (2), the parameter *A* incorporates changes due to electroporation, that is, the formation of new current pathways through cell membranes. We expect that this effect would also be influenced by temperature rise. Some studies use an alternate expression (12) to describe the contribution of the temperature rise to the conductivity (O’Brien et al., 2018, 2019):

(12) $$\sigma \left(|E,|T\right)=\sigma_{0}\cdot (1+A\cdot \mathrm{flc}2\mathrm{hs}\left(E-E_{\text{del}},E_{\text{range}}\right)\left(1+\alpha \cdot \left(T-T_{0}\right)\right)$$

In this equation, the influence of temperature rise is based on the conductivity after electroporation; however, in the present study, the temperature rise was calculated based on the initial temperature. Calculating temperature effect in this manner will overestimate the contribution of the temperature to some extent, which will make *A* decrease with pulse number within a certain pulse number range. In the present study, we calculated the temperature effect based on the initial conductivity which will consequently underestimate the effect, as part of the temperature effect was incorporated into *A*. This issue is also one of the reasons we mentioned later that it is difficult to completely decouple the temperature and the electroporation effects. However, with the conductivity model proposed in this study, the trends of *A* can still represent the cumulative effect of electroporation, leading us to choose Equation (2).

### Limitations of This Study

This study proposed a method to build the relationship between the conductivity changes of PEF-treated tissue and the pulse parameters (pulse number, electric field, and temperature) which accounts for the cumulative effects of pulses. Using the presented model, the change of the parameters *A* and *E*_0_ can be easily understood and used to explain some experimental results. Another limitation is our assumption of the often-used pulsing frequency of 1 Hz. The model might change with the pulsing frequency. However, this method might be still effective, and the effects of the pulsing frequency could be incorporated into the model. Additionally, this is an *ex vivo* study and only tested liver tissue. *In vivo* studies are needed, and more tissues need to be verified in the future. Finally, from Figure 5, at 2,500 V/cm, the numerically calculated current with our model σ(*E*, *N*, *T*) at higher pulse number was slightly larger than experimental current, but still closer than the prediction from the conventional model σ(*E*, *T*), which also can be found from the relative errors of Figure 6. This might be caused by our assumption of the tissue temperature coefficient. In the literature, α has been reported to be between 1 and 3% for soft tissue (Duck, 2013) and may vary based on tissue type and temperature. The conductivity model we used here incorporates an α value of 2%, which led to a trend in *A* which was consistent with what we would expect with cumulative electroporation effects. However, it should be noted that without knowing the exact α, our experimental setup made it impossible to completely decouple the contributions from the electroporation and thermal effects in the model. In the future, the dynamic change of temperature coefficient α should be further investigated. By incorporating these efforts, the cell death model of IRE (Golberg and Rubinsky, 2010; Garcia et al., 2014) and the multi-pulse conductivity model proposed here, the more accurate ablation zone of IRE can be predicted.

## Conclusion

In this study, the cumulative conductivity model as a function of electric field, pulse number, and temperature for rabbit liver tissue was established. By using this model, the simulated electrical currents at different pulse numbers were in good agreement with experimental results. The presented model demonstrated closer predictions of experimental current than an existing conventional conductivity model. Also, the non-linear process of electroporation can be partly described by changes in the parameters of the model. The electroporation process yields an obvious tissue conductivity change during the first few dozens of pulses (∼30) and tends to saturate. While the pulse number was considered in this study, the method proposed here can be easily transferred to investigate the effect of other parameters on dynamic conductivity changes.

## Data Availability Statement

The datasets generated for this study are available on request to the corresponding author.

## Ethics Statement

The animal study was reviewed and approved by Ethics Committee of Chongqing Medical University.

## Author Contributions

YZ, CY, and RD designed the experiments and directed the modeling process. YZ, SZ, and HL performed the experiments and analyzed the data. YZ, SZ, and NB-W established the conductivity model and ran the numerical simulation. YZ and NB prepared the manuscript. All the authors read and approved the final manuscript.

## Conflict of Interest

RD and NB-W have pending and accepted patents on IRE.

The remaining authors declare that the research was conducted in the absence of any commercial or financial relationships that could be construed as a potential conflict of interest.

## Supplementary Material

FIGURE S1 Typical current waveform during electroporation. Shown here is the response current of 1000 V which is 2000 V/cm in our study.

FIGURE S2 Dependency of *E_0*, *E_1*, and *A* with pulse number obtained by fitting the experimental data to equation (2).

FIGURE S3 *E*_*1*_ changes little with pulse number when compared with *A* and *E*_*0*_ after fitting the conductivity data to the Heaviside function for all 90 pulses. The percent changes of *E*_*0*_, *E*_*1*_, and *A* at different pulse numbers were calculated using Equation (1).

TABLE S1 Fitting results of *A* and *E*_*0*_.
