# Supplementary material for: Development of a Multi-Pulse Conductivity Model for Liver Tissue Treated With Pulsed Electric Fields
Source: Front Bioeng Biotechnol. 2020 May 19;8:396. doi: 10.3389/fbioe.2020.00396 (PMC7248411; doi:10.3389/fbioe.2020.00396)
Supplement: FIGURE S1 — Typical current waveform during electroporation. Shown here is the response current of 1000 V which is 2000 V/cm in our study. [file Data_Sheet_1.pdf]

Supplementary Material

S1: Typical current waveform during electroporation

Figure S1 is a record current waveform when the treatment voltage was 1000 V (2000 V/cm). The cell membrane typically behaves as a capacitor, and the voltage across a capacitor cannot change suddenly, which results in an overshoot in current followed by a charging process of the capacitor which will take about ~5 µs. We define this period as the capacitive current (red box in Figure S1). After that, the tissue will experience the electroporation process if the field is high enough, and the current will increase till the voltage is cut off (blue box in Figure S1). To obtain the tissue conductivity under the condition of electroporation, we chose the voltage and the current at the end of the pulse. The average values were used to eliminate the effect of noise (green box in Figure S1).

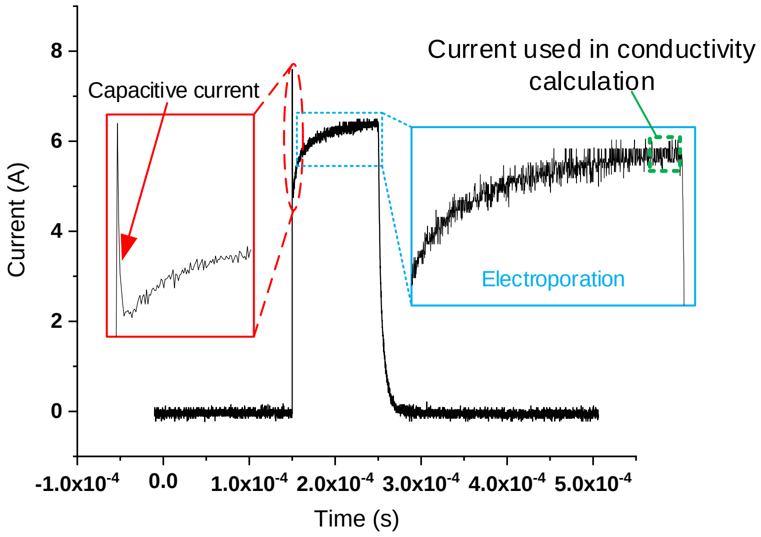

Figure S1. Typical current waveform during electroporation. Shown here is the response current of 1000 V which is 2000 V/cm in our study.

S2: The expression of the Heaviside functions, $flc2hs(E\text{-}E_{del},E_{range})$

$$flc2hs(E - E_{del}, E_{range}) = 0, \qquad if\ E - E_{del} \leq -E_{range}$$

$$flc2hs(E - E_{del}, E_{range}) = 1, \qquad if\ E - E_{del} \geq E_{range}$$

$$flc2hs(E - E_{del}, E_{range}) = 0.5 + 0.9375\left(\frac{E - E_{del}}{E_{range}}\right) - 0.625\left(\frac{E - E_{del}}{E_{range}}\right)^3 + 0.1875\left(\frac{E - E_{del}}{E_{range}}\right)^5, \quad if \ -E_{range} < E - E_{del} < E_{range}$$

S3: Relative change of the parameters in the Heaviside functions

After fitting all the conductivity data to the Heaviside function at each pulse number, the variables A, E_0, E_1 as functions of pulse number were obtained. The data were shown in Figure S2.

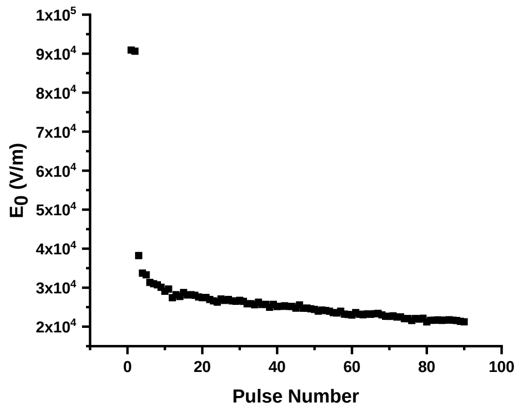

(A)

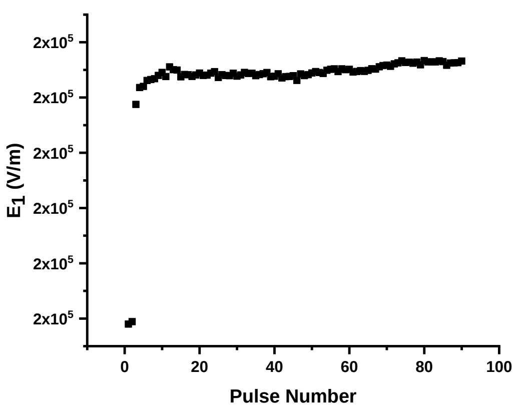

(B)

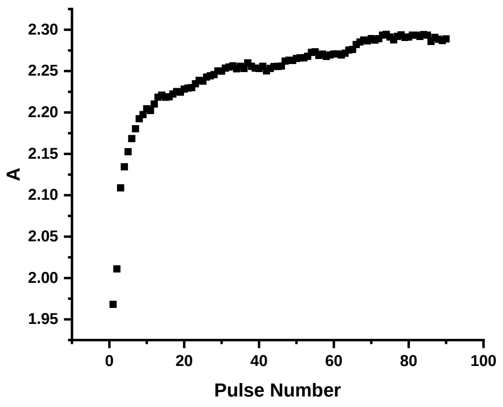

(C)

Figure S2. Dependency of E_0, E_1, and A with pulse number obtained by fitting the experimental data to equation (2).

There is a fluctuate at the first two pulses. After that, the relatively change of the of E_1 was much smaller than E_0 and A. The relatively change of these parameters was calculated using Equation (eq 1):

$$\boldsymbol{Percent\ change} = {|x_i - x_0|}/{x_0} \times 100\% \qquad \text{(eq 1)}$$

Here, x is the variable (A, E_0 or E_1), and i is the pulse number. All the variables were normalized to their value at the first pulse after the fluctuate (the third pulse).

The percent changes of these three variables are shown in **Figure S3**. The percent change of E_1 is smaller than 5%, which is much smaller than E_0 and A. Considering this, we fixed E_1 to its averaged value, which is 1937 V/cm, and fit A and E_0 to (9) and (10), respectively. After fixing E_1, the trends of E_0 and A are more smoothly than before (Figure 4).

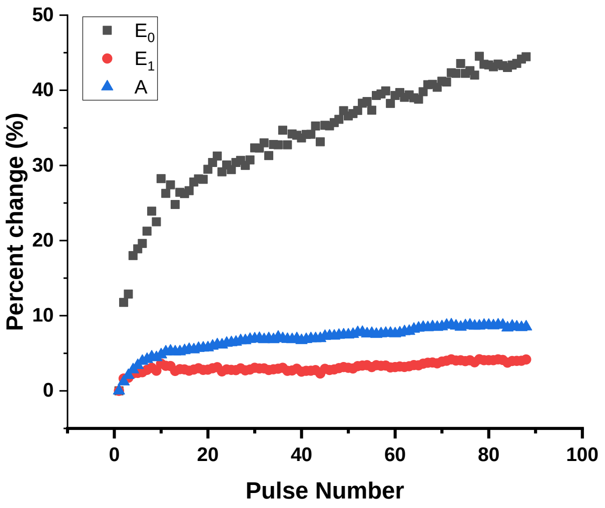

Figure S3. E_1 changes little with pulse number when compared with A and E_0 after fitting the conductivity data to the Heaviside function for all 90 pulses. The percent changes of E_0, E_1 and A at different pulse numbers were calculated using (eq 1).
